# Correlates of HIV Acquisition in a Cohort of Black Men Who Have Sex with Men in the United States: HIV Prevention Trials Network (HPTN) 061

**DOI:** 10.1371/journal.pone.0070413

**Published:** 2013-07-26

**Authors:** Beryl A. Koblin, Kenneth H. Mayer, Susan H. Eshleman, Lei Wang, Sharon Mannheimer, Carlos del Rio, Steven Shoptaw, Manya Magnus, Susan Buchbinder, Leo Wilton, Ting-Yuan Liu, Vanessa Cummings, Estelle Piwowar-Manning, Sheldon D. Fields, Sam Griffith, Vanessa Elharrar, Darrell Wheeler, for the HPTN 061 Protocol Team

**Affiliations:** 1 Laboratory of Infectious Disease Prevention, New York Blood Center, New York, New York, United States of America; 2 Fenway Health and Beth Israel Deaconess Hospital, Boston, Massachusetts, United States of America; 3 Department of Pathology, Johns Hopkins University School of Medicine, Baltimore, Maryland, United States of America; 4 Vaccine and Infectious Disease Division, Fred Hutchinson Cancer Research Center, Seattle, Washington, United States of America; 5 Department of Medicine, Harlem Hospital/Columbia University and Department of Epidemiology, Mailman School of Public Health, Columbia University, New York, New York, United States of America; 6 Department of Global Health, Emory University Rollins School of Public Health and Department of Medicine, Emory University School of Medicine, Atlanta, Georgia, United States of America; 7 Department of Family Medicine, University of California Los Angeles, Los Angeles, California, United States of America; 8 Department of Epidemiology and Biostatistics, School of Public Health and Health Services George Washington University, Washington, DC, United States of America; 9 San Francisco Department of Public Health, San Francisco, California, United States of America; 10 Department of Human Development, College of Community and Public Affairs, Binghamton University, New York, New York, United States of America; 11 Florida International University-College of Nursing and Health Sciences, Miami, Florida, United States of America; 12 FHI 360, Research Triangle Park, North Carolina, United States of America; 13 Clinical Prevention Research Branch/PSP/DAIDS/NIAID/NIH, Bethesda, Maryland, United States of America; 14 Graduate School of Social Work, Loyola University Chicago, Chicago, Illinois, United States of America; University of Mississippi Medical Center, United States of America

## Abstract

**Background:**

Black men who have sex with men (MSM) in the United States (US) are affected by HIV at disproportionate rates compared to MSM of other race/ethnicities. Current HIV incidence estimates in this group are needed to appropriately target prevention efforts.

**Methods:**

From July 2009 to October 2010, Black MSM reporting unprotected anal intercourse with a man in the past six months were enrolled and followed for one year in six US cities for a feasibility study of a multi-component intervention to reduce HIV infection. HIV incidence based on HIV seroconversion was calculated as number of events/100 person-years. Multivariate proportional hazards modeling with time-dependent covariates was used to identify correlates of HIV acquisition.

**Results:**

Of 1,553 Black MSM enrolled, 1,164 were HIV-uninfected at baseline and included in follow-up. Overall annual HIV incidence was 3.0% (95% confidence interval (CI): 2.0, 4.4%) and 5.9% among men ≤30 years old (95% CI: 3.6, 9.1%). Men ≤30 years old reported significantly higher levels of sexual risk and were more likely to have a sexually transmitted infection diagnosed during follow-up. Younger men also were more likely to not have a usual place for health care, not have visited a health care provider recently, and to have unmet health care needs. In multivariate analysis, age ≤30 years (hazard ratio (HR): 3.4; 95% CI: 1.4, 8.3) and unprotected receptive anal intercourse with HIV-positive or unknown status partners (HR: 4.1; 95% CI: 1.9, 9.1) were significantly associated with HIV acquisition.

**Conclusion:**

In the largest cohort of prospectively-followed Black MSM in the US, HIV incidence was high, particularly among young men. Targeted, tailored and culturally appropriate HIV prevention strategies incorporating behavioral, social and biomedical based interventions are urgently needed to lower these rates.

## Introduction

Men who have sex with men (MSM) comprise the largest proportion of new HIV diagnoses in the United States (US). [1] Black MSM are affected at disproportionate rates, comprising 37% of new HIV diagnoses among MSM. [2] Black MSM are three times more likely to be HIV-infected and six times more likely have an undiagnosed HIV infection compared with MSM of other race/ethnicities. [3].

Recent estimates of HIV incidence are needed to inform and monitor prevention efforts for Black MSM in the US. Most studies documenting HIV incidence among Black MSM have been based on cross-sectional studies or HIV surveillance.[2], [4]–[8] Few studies have documented HIV incidence among Black MSM from longitudinal studies and identified risk factors for incident infections.[9]–[11] The HIV Prevention Trials Network (HPTN) 061 Study (The BROTHERS Study) was designed to evaluate the feasibility of a multi-component intervention to reduce HIV incidence among Black MSM. Longitudinal data were used to estimate HIV incidence, determine correlates of HIV infection, and describe changes in sexual risk and other covariates over time in this cohort.

## Methods

### Ethics Statement

The institutional review boards (IRB) at all participating institutions approved the study: Emory University IRB #2 - Biomedical IRB (Committee A), Fenway Community Health IRB #1, University of California, Los Angeles - South General Campus IRB, Columbia University Medical Center IRB, New York Blood Center IRB, San Francisco General Hospital Committee IRB #2, and George Washington University Medical Center IRB. Written informed consent was obtained from all study participants.

### Study Sample

HPTN 061 was a multi-site study conducted in Atlanta, Boston, Los Angeles, New York City, San Francisco, and Washington, DC to determine the feasibility and acceptability of a multi-component HIV prevention intervention for Black MSM.

From July 2009 to October 2010, men were recruited directly from the community or as sexual network partners referred by index participants. Index participants were: (1) HIV infected but unaware of their infection; (2) previously diagnosed with HIV infection but not receiving HIV care and having unprotected sex with partners of negative or unknown HIV status; or (3) HIV-uninfected. Recruitment methods were developed at each site and included: community outreach; engagement of key informants and local community-based groups; print advertising; and online strategies. At each site, enrollment of community-recruited HIV-uninfected participants was capped at 200 participants; enrollment of community-recruited participants who had a prior diagnosis of HIV infection and were already in care, or reported only having unprotected anal intercourse (UAI) with HIV-positive partners, was capped at 10 participants.

Men were eligible to participate in the study if they: self-identified as a man or male at birth and as Black, African American, Caribbean Black, or multiethnic Black; were at least 18 years old; reported ≥1 instance of UAI with a man in the prior six months; resided in the metropolitan area; did not plan to move away during the time of study participation; and provided informed consent for the study. Men were ineligible if they were enrolled in any other HIV interventional research study, had been a participant in an HIV vaccine trial, or were a community-recruited participant in a category that had already reached its enrollment cap. Prescreening to determine eligibility was performed either in person or over the telephone.

### Study Visits

At the enrollment visit, eligibility was confirmed and written informed consent was obtained. Participants provided locator information and demographic information to an interviewer and then completed a behavioral assessment using audio computer-assisted self-interview (ACASI) technology. Following completion of the ACASI assessment, a social and sexual network questionnaire was completed with an interviewer. Circumcision status was determined by examination by a medical provider and if the examination was refused, status was determined by self-report.

The multi-component intervention was comprised of sexual network member referral, HIV and sexually transmitted infection (STI) testing/counseling and referral for care, and the opportunity to work with a Peer Health Navigator (PHN) who assessed service needs and developed an action plan with the participant. The ACASI, social and sexual network questionnaire, HIV and STI testing and counseling were repeated 6 and 12 months after enrollment.

### Interviewer-administered Questions

Demographic characteristics, collected by an interviewer, included age, sexual identity, education, employment, household income, and student status. Health-care related information included health care coverage, usual place of care, visits to a health care provider in the prior 6 months, and unmet health care needs in the prior 6 months.

### ACASI-administered Questions

The ACASI interview collected data on HIV testing history, history of incarceration, sexual identity and sexual risk behaviors in the 6 months prior to enrollment, including number of male, female and transgender partners; number of new partners; HIV status and race/ethnicity of partners; partner type; number of receptive and insertive anal sex acts and use of condoms; and exchange of sex for money, drugs or goods. Questions related to sex acts were asked for the last sex episode with a male partner, primary male partner, and other male partners separately.

Measures for alcohol use frequency, amount and dependency were derived from the Alcohol Use Disorders Identification Test (AUDIT) [12]; a score of >8 was used to indicate an alcohol problem. Questions on other substance use in the 6 months prior to enrollment included use of marijuana; inhaled nitrates; smoked and powder cocaine; methamphetamine; heroin; non-prescribed Vicodin, Oxycontin, or Xanax; Viagra, Cialis, or Levitra; hallucinogens and injection drug use. A variable for stimulant use in the prior 6 months was created, based on answers to use of smoked and powder cocaine and methamphetamine.

Perceived racism and sexual discrimination were measured with 28 and 25 items, respectively, such as the occurrence of “being treated rudely or disrespectfully” because of “my race” and “my sexuality”. [13] The extent that the event bothered the participant was measured with a 5-point scale from “not at all” to “extremely” (α = 0.95 for race; α = 0.95 for sexuality). The sum of scores for the racism scale was from 0 to 140; the variable was categorized as “Never happened/Low” (score from ≤47), “Medium” (score from 48–94) and “High” (score ≥95) level of experiences of racism. For the sexual discrimination scale, the sum of scores was from 0 to 125; the variable was categorized as “Never happened/Low” (score from ≤42), “Medium” (score from 43–84) and “High” (score ≥85) level of experiences of sexual discrimination.

Internalized homophobia was measured with a 7-item, 5-point Likert-type scale adapted from Herek and Glunt [14] with responses ranging from “disagree strongly” to “agree strongly” (α = 0.91). The scale included items such as “In the past 90 days, I have tried to stop being attracted to men” and “As a Black man, I try to act more masculine to hide my sexuality”. The sum of scores was categorized as having low (score ≤16) or medium (score from 17–26) or high internalized homophobia (score ≥27).

The Center for Epidemiologic Studies Depression Scale (CES-D) was used to measure depression. [15] Items such as on how many days in the past week “I was bothered by things that usually don't bother me” were answered using a 20-item, 4-point scale of “less than 1 day (Rarely/none of the time)” to “5–7 days (Most of the time)”. Using the sum of scores, a score of 16 or higher was indicative of depressive symptoms.

Social Support was measured with 6 items such as “How often is there someone available to whom you can count on to listen to you when you need to talk?” with responses on a 5-point scale from “none of the time” to “all the time” (α = 0.94). Using the sum of scores, a participant was categorized as having low social support (sum score ≤13), or median support (14<sum≤21), or high support (sum≥22). [16].

Internalized HIV stigma was measured with 5 items such as “Society looks down on people who have HIV” with responses on a 5-point scale from “disagree strongly” to “agree strongly” (α = 0.82). [17] Using the sum of scores, a participant was categorized as having low (score ≤15) or high (score >15) internalized HIV stigma.

### Laboratory Testing

HIV rapid testing was conducted after completion of interviews and pre-test HIV/STI risk-reduction counseling. Reactive rapid HIV test results were confirmed by Western blot testing performed at a local laboratory. Quality assurance testing was performed retrospectively at the HPTN Network Laboratory to confirm the HIV infection status of all study participants at enrollment and to confirm cases of HIV seroconversion (excluding those with acute infection at enrollment). Enrollment samples with low or undetectable HIV RNA were retrospectively tested for antiretroviral drugs to identify men on antiretroviral treatment who did not report a prior HIV diagnosis. Urine and rectal swabs were collected for Neisseria gonorrhea and Chlamydia trachomatis testing and a blood specimen was collected for syphilis testing. All participants who had a positive test for any STI were referred for treatment and medical and social services, as needed.

### Statistical Analysis

Frequency distribution of demographics and baseline characteristics (including sexual risk behaviors, substance use, STIs) were summarized across study cities. For occurrence of an STI, a dichotomized variable (any vs. none) was derived for each participant at each study visit based on urine and rectal test results for gonorrhea and Chlamydia and syphilis test results.

Incident rates were based on HIV seroconversions during study follow-up with person-year analysis. Exact Poisson-based confidence intervals were computed. We assumed that HIV serostatus was negative at a missing visit if there were no positive results at earlier visits and an assessment was made at a subsequent visit.

To identify correlates of HIV acquisition, we first conducted univariate Cox proportional hazards regression for each covariate. The variables included fixed covariates (city, age, sexual identity, and circumcision status) and time-dependent covariates (STIs, number of male partners, gender of partners, primary partner, new partner, race/ethnicity of partners, unprotected receptive (URAI) and insertive anal intercourse (UIAI), exchange of sex, alcohol dependency and use of stimulants). Covariates that were significant at p<0.05 in the univariate model were then considered in a multivariate proportional hazards model. The behavioral variables were assessed at the same time that the blood was drawn for HIV testing, and referred to the previous 6-month time period. Additionally, since the behavioral variables were time-varying, the values of these covariates could change from one 6-month interval to the next. To accommodate missing data, we applied the last-value carried forward convention to the time-varying covariates except for indicators for STIs.

We further compared the socioeconomic factors, health care utilization, sexual behaviors, psychosocial factors and structural factors between younger (age: 18–30 years) and older (>30 years) participants. Baseline characteristics were compared by Chi-Square test. A generalized estimating equations approach for logistic regression was used to assess differences in risk behavior change by age group. This method accounts for within-subject variation over time. Data were censored at the visit where a participant first tested HIV positive during study follow-up. Any of these variables found to be significantly associated with age and HIV incidence were considered for inclusion in the multivariate model of HIV incidence. SAS® version 9.2 statistical software was used to perform all analyses.

## Results

### Study Population

**Figure 1:**
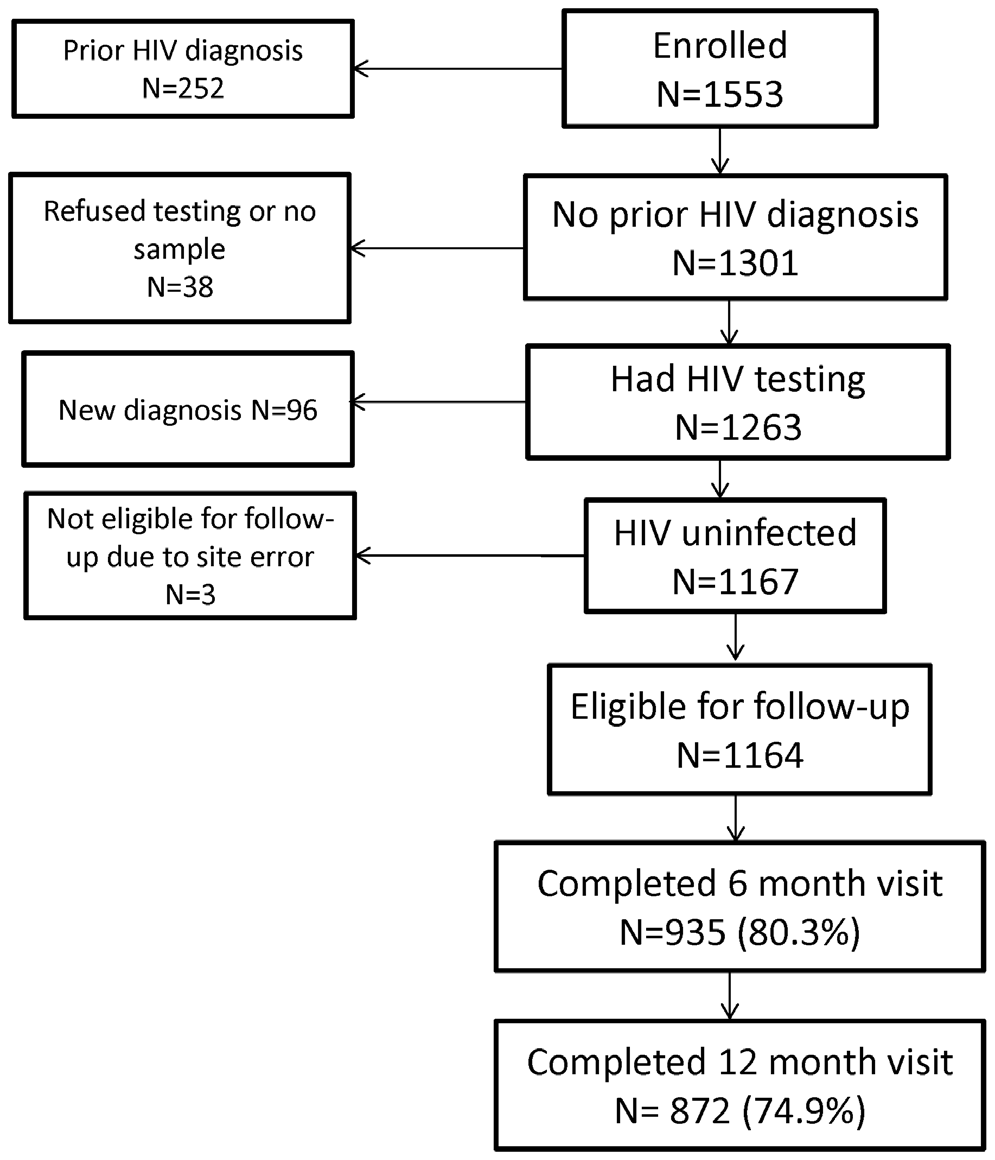
Flow diagram of study sample, HPTN 061*. *Men were categorized as previously diagnosed (prior HIV diagnosis) based on self-report or retrospective detection of antiretroviral drugs in samples from the enrollment visit (see Methods). The newly diagnosed group included three men who had acute HIV infection at enrollment.

Of 1,553 Black MSM enrolled, 1,301 did not have a prior HIV diagnosis at baseline. Of those, 1,263 (97.1%) had an HIV test and 1,164 were HIV-uninfected at baseline and were included in follow-up (Figure 1). The mean age of the HIV-uninfected men was 37.0 years with 37.9% between 18 and 30 years (Table 1). About one-quarter (26.3%) of men identified as homosexual or gay, while another 30.5% identified as bisexual. Almost one-half (45.8%) of men had at least some college education. Annual income was low; 58.3% reported an income of less than $20,000 and 65.3% were not working.

**Table 1:** Demographic characteristics and baseline behaviors of HIV-uninfected Black MSM, HPTN 061 (n = 1164).

Characteristic		N*	%
City	Atlanta	224	19.2
	Boston	191	16.4
	Los Angeles	189	16.2
	New York	221	19.0
	San Francisco	174	14.9
	Washington, DC	165	14.2
Age	18–30	441	37.9
	31+	723	62.1
Sexual identity	Homosexual/Gayonly	300	26.3
	Bisexual only	348	30.5
	Other	492	43.2
Education	Less than college	630	54.2
	Some college ormore	533	45.8
Household income	<$10,000	438	38.1
	$10,000–19,999	233	20.2
	$20,000–29,000	148	12.9
	≥$30,000	332	28.8
Employment status	Full-time	178	15.3
	Part-time	225	19.3
	Not working	760	65.3
Student status	Part or full-timestudent	247	21.2
	Non-student	917	78.8
Circumcision status	Circumcised	881	75.8
	Uncircumcised	281	24.2
*In prior 6 months:*			
Sexually transmitted infections**		157	13.5
No. of male partners	0–1	243	20.9
	2–4	559	48.1
	5+	359	30.9
Gender of partners	Male only	614	54.0
	Male and female	522	46.0
Had a primary male partner		541	47.6
Had a new male partner		973	86.0
Race/ethnicity of male partners	All Black	584	51.1
	Some Black	366	32.0
	None	192	16.8
URAI		538	46.9
URAI with HIV+/unknown status partners		285	26.1
URAI with HIV negative partners		316	27.8
UIAI		874	75.9
UIAI with HIV+/unknown status partners		503	46.1
UIAI with HIV negative partners		524	46.2
Received money/goods for sex		345	29.9
Provided money/goods for sex		188	16.3
Alcohol problem (AUDIT score >8)		378	41.9
Stimulant use		425	38.0
Stimulant use with UAI		288	29.2
Marijuana use		640	56.6

* N may not add to total due to missing values.

** based on study testing for syphilis and urethral/rectal gonorrhea and Chlamydia. URAI = unprotected receptive anal intercourse; UIAI =  unprotected insertive anal intercourse.

Using clinical examination and self-report for those without an exam (44.9% of men), 24.2% were uncircumcised. At least one STI was diagnosed in 13.5% of men at baseline. URAI was reported by 46.9% of men at baseline and UIAI by 75.9%. Stimulant use was reported by 38.0% of men (18.9% cocaine, 25.7% crack and 9.6% methamphetamines) and marijuana use by 56.6% (Table 1). All other drugs were reported by 10% or less of men at baseline.

### Retention

Study visits were completed by 80.3% of men at 6 months and 74.9% at 12 months (Figure 1). HIV infection status was determined for 86.5% of men at the 6-month visit and 76.5% of men at the 12-month visit. No differences were found between men who did not complete any follow-up visits and men who had at least one follow-up visit with regard to demographics, circumcision status, unprotected intercourse, exchange of sex for money or goods, occurrence of STIs or substance use (data not shown).

### HIV Incidence and Correlates

During follow-up, 28 men acquired HIV infection over 926 person-years of follow-up, for a 3.0% annual HIV incidence rate (95% CI: 2.0, 4.4%). The HIV incidence rates were 3.7 per 100 person-years (95% CI: 2.0, 5.4%) during months 0–6 and 2.2 per 100 person-years (95% CI: 0.9, 3.4%) during months 6–12. In univariate analyses, several factors were significantly associated with higher HIV incidence: residence in Los Angeles compared to Boston; age 18 to 30 years compared to older ages; gay/homosexual identity compared to bisexual, diagnosis with an STI during the study, reporting 2+ male partners in the prior 6 months, and reporting URIA with HIV-positive or unknown status male partners in the prior 6 months (Table 2). Alcohol dependency was associated with a lower HIV incidence (Table 2). In the multivariate model, only younger age and URAI with HIV-positive or unknown status partners remained statistically significant (Table 2). Considering age as a continuous variable did not change the results.

**Table 2:** HIV incidence rates with time-dependent Cox proportional hazard models among Black MSM, HPTN 061.

Characteristic		No. HIVinfections	Person-years	HIV incidenceper 100 p-yrs	Unadjusted HR(95% CI)	Adjusted HR(95% CI)
City	Boston	1	156.6	0.6	REF	REF
	New York	2	186.9	1.1	1.6 (0.1, 17.8)	1.3 (0.1, 14.3)
	Washington, DC	3	123.5	2.4	3.5 (0.4, 33.6)	1.5 (0.2, 15.3)
	San Francisco	4	148.1	2.7	4.3 (0.5, 38.3)	4.3 (0.5, 39.4)
	Atlanta	8	166.7	4.8	7.3 (0.9, 58.2)	6.2 (0.8, 50.5)
	Los Angeles	10	144.1	6.9	10.2 (1.3, 80.0)	5.0 (0.6, 41.1)
Age	18–30	20	339.7	5.9	4.3 (1.9, 9.7)	3.4 (1.4, 8.3)
	31+	8	586.3	1.4	REF	REF
Sexual identity	Bisexual only	4	273.5	1.5	REF	REF
	Other	12	396.0	3.0	2.0 (0.7, 6.3)	1.0 (0.3, 3.3)
	Homosexual/Gay only	12	238.0	5.0	3.4 (1.1, 10.6)	1.5 (0.5, 4.9)
Circumcision status	Circumcised	21	699.1	3.0	REF	
	Uncircumcised	7	224.9	3.1	1.1 (0.4, 2.5)	–
*In prior 6 months:*						
Sexually transmitted infection	No	22	800.0	2.8	REF	REF
	Yes	6	84.3	7.1	2.5 (1.0, 6.3)	1.7 (0.6, 4.7)
No. male partners	0–1	7	431.1	1.6	REF	REF
	2+	21	45.0	4.2	2.6 (1.1, 6.1)	1.6 (0.7, 3.9)
Gender of partners	Male only	22	468.4	4.7	2.6 (0.98, 6.9)	–
	Male and female	5	285.6	1.8	REF	
Had primary male partner	No	19	498.9	3.8	1.8 (0.8, 3.9)	–
	Yes	9	419.7	2.1	REF	
Had a new male partner	No	6	267.2	2.2	REF	–
	Yes	22	650.6	3.4	1.5 (0.6, 3.7)	
Race/ethnicity of partners	All Black	18	491.3	3.7	2.3 (0.7, 7.9)	–
	Some Black	7	236.6	3.0	1.9 (0.5, 7.4)	
	None Black	3	193.5	1.6	REF	
URAI with HIV+/unk status partners	No	16	787.9	2.0	REF	REF
	Yes	12	118.6	10.1	5.0 (2.4, 10.6)	4.1 (1.9, 9.1)
URAI with HIV negative partners	No	21	739.2	2.8	REF	
	Yes	7	183.6	3.8	1.3 (0.6, 3.1)	–
UIAI with HIV+/unk status partners	No	18	693.5	2.6	REF	
	Yes	10	213.1	4.7	1.8 (0.8, 4.0)	–
UIAI with HIV negative partners	No	22	631.9	3.5	REF	
	Yes	6	290.9	2.1	0.6 (0.2, 1.5)	–
Received money/goods for sex	No	25	772.1	3.2	REF	
	Yes	3	153.4	2.0	0.6 (0.2, 2.0)	–
Alcohol problem (AUDIT score >8)	No	22	516.4	4.3	REF	REF
	Yes	4	286.7	1.4	0.3 (0.1, 1.0)	0.4 (0.1, 1.1)
Stimulant use	No	20	627.6	3.2	REF	
	Yes	8	295.1	2.7	0.9 (0.4, 1.9)	–
Stimulant use with UAI	No	23	674.1	3.4	REF	
	Yes	5	201.1	2.5	0.7 (0.3, 1.9)	–

HR =  hazard ratio; REF =  reference group: URAI = unprotected receptive anal intercourse; UIAI =  unprotected insertive anal intercourse; UAI =  unprotected anal intercourse.

### Changes in Behaviors and STIs and Differences by Age Group

**Figure 2:**
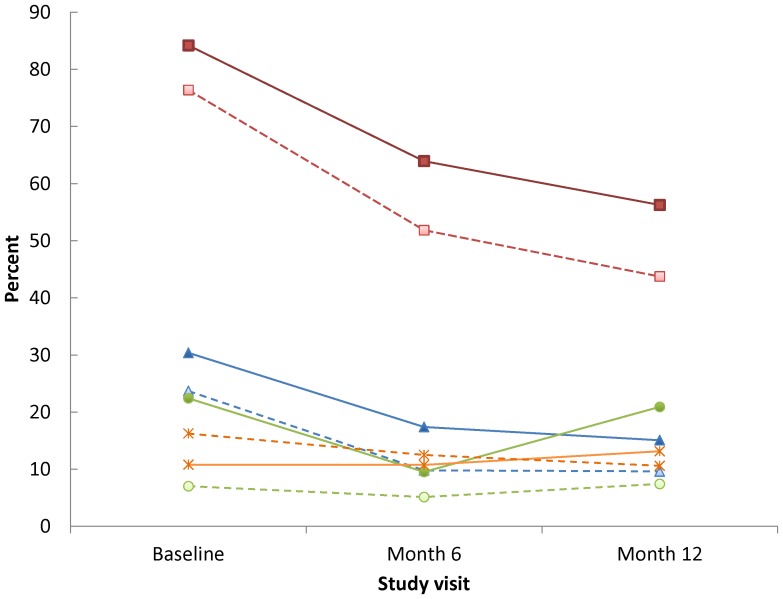
Percent with sexually transmitted infections and self-reported risk behaviors by study visit and age group. Solid lines ≤30 years of age, dashed lines >30 years of age. Red squares: 2+ male partners; Blue triangles: Unprotected receptive anal intercourse with positive or unknown status male partners; Green circles: Sexually transmitted infections; Orange stars: HIV- positive male partners.

A significant decline was observed in the proportion of men reporting two or more male partners from enrollment to the 12-month follow-up visit (OR = 0.2; 95% CI: 0.2, 0.3) (Figure 2). During this time period, men ≤30 years of age were significantly more likely to report two or more partners (OR = 1.6; 95% CI: 1.3, 2.0) compared to older men. Declines also were observed in the proportion of men reporting URAI with HIV-positive or unknown status partners from enrollment to the 6-month visit (OR = 0.4; 95% CI: 0.3, 0.5); no significant change was seen between the 6- and 12-month visits (OR = 0.9; 95% CI: 0.7, 1.2). Younger men were more likely to report URAI with HIV-positive or unknown status partners (OR = 1.5; 95% CI: 1.2, 2.0) compared to older men. Overall, the proportion of men reporting an HIV-positive male partner did not significantly change during follow-up (OR = 0.9; 95% CI: 0.7, 1.1) and there were no significant differences by age group (OR = 0.9; 95% CI: 0.6, 1.2). Finally, the occurrence of STIs declined from baseline to the 6-month visit (OR = 0.5; 95% CI: 0.4, 0.7), but then increased by the 12-month visit (OR = 2.0; 95% CI: 1.5, 2.6) (Figure 2). The proportion of younger men with an STI was significantly higher compared to older men (OR = 3.3; 95% CI: 2.4, 4.5). Inclusion of occurrence of STIs, number of male partners, and URAI with HIV-positive or unknown status partners along with study site, identity, and alcohol dependency reduced the HR for younger age somewhat from 4.3 (95% CI: 1.9, 9.7) to 3.4 (95% CI: 1.4, 8.3) (Table 2).

We also examined differences in socioeconomic factors, health care utilization and access, psychosocial variables, and structural factors by age group. Younger men were significantly more likely to have a higher income, to be employed and to have a higher educational level (Table 3). No significant differences were found with regards to having health insurance, but younger men were more likely to report not having a usual place of care. During follow-up, younger men were more likely to report not having seen a health care provider in the prior 6 months (OR: 1.3; 95% CI: 1.1, 1.6) and to report needing health care but not being able to get it (OR: 1.4; 95% CI: 1.1, 1.9). During follow-up, younger men also were more likely to score high on the internalized HIV stigma scale (OR: 1.4; 95% CI: 1.1, 1.7) but less likely to have a low level of social support (OR: 0.6; 95% CI: 0.5, 0.8) and high level of internalized homophobia (OR: 0.5; 95% CI: 0.4, 0.7). No significant differences were found in reporting of depressive symptoms by age group. Younger men were significantly less likely to become incarcerated during follow-up (OR: 0.4; 95% CI: 0.3, 0.5). No significant differences were found in reporting of experiences of racism or sexual discrimination by age group. None of the variables described above were significantly associated with HIV acquisition and thus were not included in the multivariate model.

**Table 3:** Baseline characteristics by age group, HPTN 061.

		18–30 years		31+ years		
		N	%	N	%	p-value
City	Atlanta	53	13.9	130	20.7	<0.01
	Boston	49	12.9	115	18.3	
	Los Angeles	69	18.2	98	15.6	
	New York	75	19.7	126	20.1	
	San Francisco	35	9.2	113	18.0	
	Washington DC	99	26.1	46	7.3	
Socioeconomic factors						
Household income	<$10,000	131	35.0	241	38.6	<0.01
	$10,000–19,999	60	16.0	143	22.9	
	$20,000–29,000	48	12.8	86	13.8	
	≥$30,000	135	36.1	154	24.7	
Employment status	Full-time	76	20.0	80	12.8	<0.01
	Part-time	101	26.6	95	15.2	
	Not working	203	53.4	452	72.1	
Education	Less than college	178	47.0	363	57.8	<0.01
	Some college+	201	53.0	265	42.2	
Health care related factors						
Ever tested for HIV		357	93.9	573	91.4	0.14
Tested for HIV in last year		335	88.2	500	80.9	<0.01
Had health insurance		223	58.7	387	61.6	0.35
Usual place of care*	None	116	30.5	85	13.5	<0.01
	Community health center/clinic	101	26.6	225	35.8	<0.01
	Hospital outpatient clinic	57	15.0	134	21.3	0.01
	Emergency room	52	13.7	158	25.2	<0.01
	Private doctor’s office	61	16.1	69	11.0	0.02
	All other	37	9.7	70	11.1	0.48

* more than one option allowed.

### Uptake of Intervention Components and HIV Incidence

Three intervention components were included in this analysis: uptake of STI testing, utilization of peer health navigation, and referral of a sexual partner. During follow-up, 816 (70.1%) men agreed to STI testing only, 294 (25.3%) agreed to STI testing and utilized peer health navigation, 35 (3.0%) agreed to STI testing and referred a sexual partner and 18 (1.6%) agreed to STI testing, utilized peer health navigation and referred a sexual partner. Among men agreeing to STI testing only, the annual HIV incidence rate was 2.9% (95% CI: 1.7, 4.6) and among men agreeing to STI testing and utilizing peer health navigation, the annual HIV incidence rate was 3.1% (95% CI: 1.4, 6.2). The number of men in the other groups was too small to calculate HIV incidence.

## Discussion

In the largest longitudinal cohort of Black MSM enrolled to date in the US, a high annual HIV incidence was found, with the highest incidence (almost 6%) among young Black MSM. The CDC estimates that 4,800 new HIV infections occurred among young Black MSM (aged 13–24 years) in 2010, which is 2.7 times higher than the number among young White MSM. New infections among young Black MSM compromised 45% of new infections among Black MSM [18]. Based on extrapolations developed by Stall et al [19], a 4.0% annual incidence rate among an HIV-uninfected cohort of 18 year old MSM would result in 25% of these men being HIV infected by age 25 and 50% being HIV infected by age 30; this further illustrates the urgency associated with the HIV incidence rates identified in the HPTN 061 cohort. HIV incidence in this cohort could have been over-estimated due enrollment higher risk men. Alternatively, HIV incidence may have been under-estimated in this cohort, since participants were offered HIV prevention components, including HIV testing and STI testing and referral for STI and HIV treatment, peer counseling, and referral for health care and other supportive services.

The proportion of younger men with STIs was more than three times that among older men; a higher proportion of younger men also reported sexual risk behaviors compared to older men. Issues of health care access and use were more prevalent among younger men compared to older men. The combination of higher sexual risk behaviors, occurrence of STIs known to increase the risk of HIV acquisition [20], [21], and health care access issues may be significant drivers of HIV acquisition among young Black MSM [3]. At the same time, younger men reported higher incomes, lower prevalence of incarceration, high levels of social support and lower levels of internalized homophobia. These findings suggest that prevention strategies need to be mindful of the influence of differing socioeconomic classes of young Black MSM who have “come out” in a post gay-liberation movement.

It is important to note that several factors were not associated with HIV incidence in this cohort, namely, circumcision status, having predominately Black male partners, and stimulant use. A review of the literature on the potential for a protective effect of circumcision for MSM suggested that circumcision may be protective for MSM who are predominately insertive during anal intercourse, but not for MSM who are receptive [22]. In this cohort, three-quarters of men practiced UIAI, but over half also engaged in URAI, thus likely limiting any effect of circumcision. As reported in the literature, Black MSM are significantly more likely to have Black sex partners compared to men of other race/ethnicities [3], [23], suggesting that the disparity in HIV infection by race/ethnicity could be explained by a higher HIV prevalence among sexual partners. In this study, however, having all or some Black male sex partners was not significantly associated with HIV incidence. Finally, previous cohorts of MSM have demonstrated a significant increase in HIV risk associated with stimulant use, predominately due to methamphetamine use. [10], [24] Although over one-third of men reported stimulant use, this use was not associated with risk of HIV acquisition, perhaps due to cocaine as the stimulant drug of choice, rather than methamphetamines.

Overall, reporting of multiple male sex partners and URAI with positive and/or unknown status partners declined significantly among the men during the study. HIV incidence also declined from 3.7% to 2.2% between 0–6 and 6–12 months, but this change was not statistically significant. In contrast to the reported sexual risk behavior, the occurrence of STIs initially declined between enrollment and the 6-month visit, but then increased by a factor of two in the last 6 months of follow-up. These results emphasize the complexity of the relationship between reported risk behaviors and non-HIV biologic outcomes in determining HIV exposure.

There are several limitations to this study that should be recognized. First, this cohort does not represent Black MSM in the US. The eligibility criteria included a report of recent unprotected sex, the overall socioeconomic status of the men was relatively low and the study was conducted in six US cities with high HIV prevalence. Second, the final retention rate at 12 months was not optimal. However, we did not observe any systematic difference between men who were retained compared to men who were not retained. Finally, all variables, except occurrence of STIs and HIV acquisition, were self-reported and may have been under-reported, though ACASI was used to try to minimize social desirability bias.

In conclusion, our findings underscore the importance of incorporating a multi-factorial approach when addressing the HIV epidemic among Black MSM in the US. To address high HIV incidence among Black MSM, culturally-relevant prevention interventions addressing psychosocial factors and structural barriers are needed that integrate HIV and STI prevention and treatment.
